# Ocular Dirofilariasis in Migrant from Sri Lanka, Australia

**DOI:** 10.3201/eid3004.240125

**Published:** 2024-04

**Authors:** Elliott D. Cope, Nishant Gupta, Anson V. Koehler, Robin B. Gasser, Amy Crowe

**Affiliations:** Royal Victorian Eye and Ear Hospital, East Melbourne, Victoria, Australia (E.D. Cope, N. Gupta);; The University of Melbourne, Parkville, Victoria, Australia (A.V. Koehler, R.B. Gasser);; St Vincents Hospital, Fitzroy, Victoria, Australia (A. Crowe)

**Keywords:** ocular dirofilariasis, *Dirofilaria*, Hong Kong genotype, parasites, Australia, Sri Lanka, zoonoses, nematodes, vector-borne infections

## Abstract

We describe a case of imported ocular dirofilariasis in Australia, linked to the Hong Kong genotype of *Dirofilaria* sp., in a migrant from Sri Lanka. Surgical extraction and mitochondrial sequences analyses confirmed this filarioid nematode as the causative agent and a *Dirofilaria* sp. not previously reported in Australia.

Human dirofilariasis, caused by nematodes such as *Dirofilaria repens* or *D. immitis*, is a zoonotic disease transmitted through the bite of various mosquito species (*1*). The definitive hosts of *Dirofilaria* sp. nematodes are canine and feline populations. Clinical manifestations of human infections, including the development of nodules in multiple anatomic locations, result from the migration or dwelling of worms in subcutaneous tissues (*1*). Human dirofilariasis is typically species-specific depending on the geographic area; *D. repens* nematode infections are found in Europe and Asia and *D. immitis* nematode infections in the Americas (*1*). Recently, a new genotype, called *Dirofilaria* sp. *hongkongensis* in the literature and referred to in this article as *Dirofilaria* sp. Hong Kong genotype (*2*), is proposed as a causative agent of subcutaneous or subconjunctival dirofilariosis in humans with a likely reservoir in canines. *Dirofilaria* sp. Hong Kong genotype is considered a nomen nudum within the scientific community because a proper morphologic description is missing (*3*). In Australia, only 21 human cases of dirofilariasis have been reported (*1*). Four documented cases of orbital dirofilariasis have been linked to suspected *Drofilaria* infection (*1*).

*Dirofilaria* nematode infection is usually transmitted by mosquitoes (including those of the *Aedes*, *Anopheles*, and *Culex* genera) from carnivores. Mosquitoes play a crucial role in transmission by injecting the microfilaria into the accidental human host, which enables the transmitted larvae to develop further, but the nematodes do not typically reach full maturity in the human host, and they are usually sequestered in tissue. In rare instances, the adult stages of *Dirofilaria* nematodes are found in humans, usually in the lungs or in the cutaneous or subconjunctival areas (*1*).

In Australia, estimated prevalence of the *D. immitis* nematode in the canine population is 20% in some areas of the east coast, home to many species and genera of mosquitoes (e.g., *Aedes*, *Anopheles*, and *Culex*) (*4**,**5*,*6*). Of the 21 human dirofilariasis cases reported from mainland Australia in the past 40 years, most are linked to *D. immitis* nematode infection, and others are linked to *D. repens* nematode infection in returned travelers (*1*,*4*). We describe an imported case of human dirofilariasis of the Hong Kong genotype in Australia.

A 77-year-old man with a history of heart disease and diabetes was referred to the emergency department of the Royal Victorian Eye and Ear Hospital in Melbourne, Victoria, Australia, by an ophthalmologist because of concerns about a possible worm in the subconjunctiva. The patient had redness and pain in his left eye for 1 month but reported no visual impairment. He had immigrated from Sri Lanka to Melbourne 18 months earlier, and he had no history of recent travel, pet ownership, freshwater swimming, or gardening. Our examination of his left eye revealed normal visual acuity (6/6) and intraocular pressures (14 mm Hg). During the examination, we observed a mobile, whitish, curled, translucent, and elongated foreign body in the subconjunctiva positioned at approximately 4 o’clock, adjacent to the limbus, with an overlying mild conjunctival injection. Our posterior segment assessment of the eye (dilated pupil) was unremarkable (Figure 1). We surgically removed the foreign body, which was a 12-cm-long worm (Figure 2), after a limited peritomy (Appendix Figure). We then suspended the worm in physiologic saline and submitted it for macroscopic and genetic analysis. The patient was discharged the next day and returned for a follow-up examination 1 week later. 

**Figure 1:**
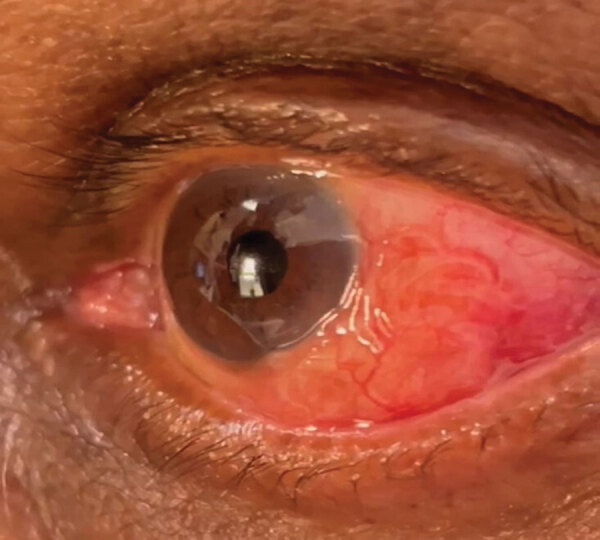
Left eye of a patient who recently migrated to Australia and originated from Sri Lanka, showing a subconjunctival infection that was identified as *Dirofilaria* sp. Hong Kong genotype nematode. The nematode can be seen at 3–5 o’clock, adjacent to the limbus of the eye.

**Figure 2:**
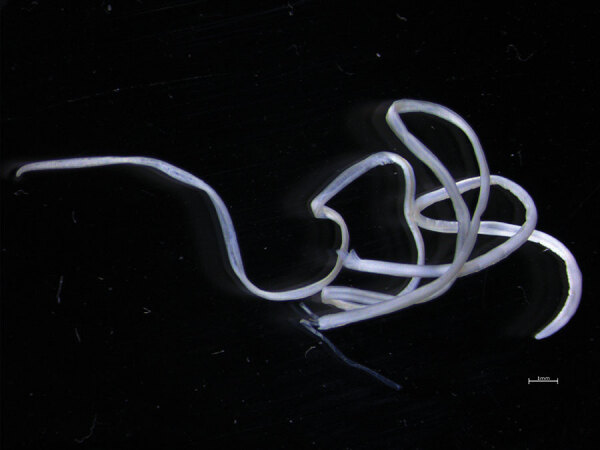
Macroscopic view of a *Dirofilaria* sp. Hong Kong genotype nematode after surgical extraction from the left eye of a patient who recently migrated from Sri Lanka to Australia. The nematode was 12 cm long. Scale bar indicates 1 mm.

Our microscopic examination of the surgically removed worm confirmed that it was a female worm with a thick, nonridged cuticle and a complete alimentary tract and reproductive system. We found no larvae in the uterus. We genetically characterized the worm by using PCR-based sequencing of a portion of the mitochondrial cytochrome c oxidase 1 (941 bp) gene and of the 12S nuclear ribosomal RNA gene (610 bp) (*7*). The sequences we obtained (GenBank accession nos. OR755977 and OR768484) were almost identical (940/941 bp for mitochondrial cytochrome c oxidase 1; 610/611 bp for 12S) to those representing the Hong Kong genotype of the *Dirofilaria* nematode (GenBank accession no. KX265050).

We presume that the patient acquired the *Dirofilaria* nematode infection in Sri Lanka, where prevalence of dirofilariasis is high (30%–69%) in feline and canine populations and some cases are linked to the Hong Kong genotype (*8*). Of the 173 reported cases of human dirofilariasis caused by *D. repens* nematodes reported during 1965–2020, a total of 40 cases were in patients with subconjunctival infections (*9*). We are concerned that some of those infections might have been misidentified as *D. repens* because molecular methods were not used for genetic analysis and identification (*8*).

This study emphasizes the importance of using molecular tools for the accurate diagnosis of filariases and the need for heightened clinical suspicions of rare zoonotic infections. This emphasis is particularly important for patients who have a travel history from countries endemic for neglected tropical diseases.

Appendix Additional information about case of ocular dirofilariasis in migrant from Sri Lanka, Australia.
