# Supplementary material for: Ocular Dirofilariasis in Migrant from Sri Lanka, Australia
Source: Emerg Infect Dis. 2024 Apr;30(4):829–30. doi: 10.3201/eid3004.240125 (PMC10977853; doi:10.3201/eid3004.240125)
Supplement: Appendix — Additional information about case of ocular dirofilariasis in migrant from Sri Lanka, Australia. [file 24-0125-Techapp-s1.pdf]

Article DOI: https://doi.org/10.3201/eid3004.240125

EID cannot ensure accessibility for supplementary materials supplied by authors. Readers who have difficulty accessing supplementary content should contact the authors for assistance.

Ocular Dirofilariasis in Migrant from Sri Lanka, Australia

Appendix

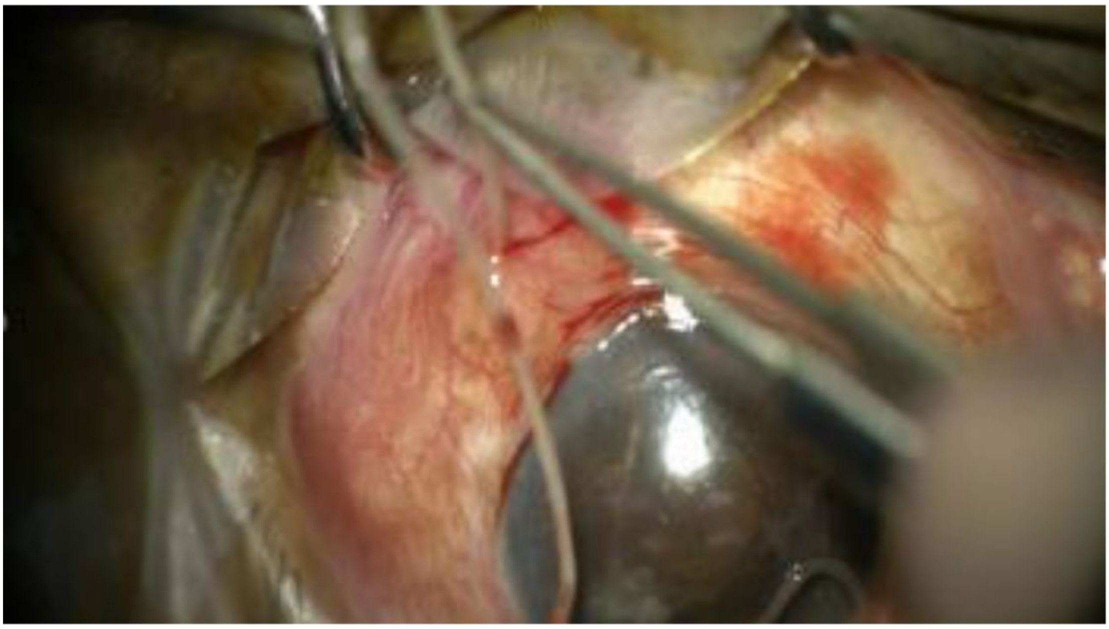

Appendix Figure. Surgical removal of subconjunctival infection by *Dirofilaria* sp. 'Hong Kong genotype' from a patient in Australia who recently migrated from Sri Lanka.
